# Oral health-related quality of life and related factors among residents in a disaster area of the Great East Japan Earthquake and giant tsunami

**DOI:** 10.1186/s12955-015-0339-9

**Published:** 2015-09-15

**Authors:** Mitsuo Kishi, Fumie Aizawa, Miki Matsui, Yukari Yokoyama, Akiko Abe, Kentaro Minami, Ruriko Suzuki, Hiroyuki Miura, Kiyomi Sakata, Akira Ogawa

**Affiliations:** Division of Preventive Dentistry, Department of Oral Medicine, School of Dentistry Iwate Medical University, 1-3-27 Chuo-dori, Morioka, Iwate 020-8505 Japan; Department of Social Welfare, Nihon Fukushi University, Okuda, Mihama Town, Aichi 470-3295 Japan; Faculty of Community Nursing, Iwate College of Nursing 14-1 Sengakubo, Ogama, Takizawa, Iwate 020-0751 Japan; Division of Orthodontics, Department of Developmental Oral Health Science, School of Dentistry Iwate Medical University, 1-3-27 Chuo-dori, Morioka, Iwate 020-8505 Japan; Department of Hygiene and Preventive Medicine, School of Medicine Iwate Medical University, 2-1-1 Nishitokuta, Yahaba, Iwate 028-3694 Japan; Iwate Medical University, 19-1 Uchimaru, Morioka, Iwate 020-8505 Japan

## Abstract

**Background:**

Oral health is one of the most important issues for disaster survivors. The aim of this study was to determine post-disaster distribution of oral health-related quality of life (OHRQoL) and related factors in survivors of the Great East Japan Earthquake and Tsunami.

**Methods:**

Questionnaires to assess OHRQoL, psychological distress, disaster-related experiences, and current systemic-health and economic conditions were sent to survivors over 18 years of age living in Otsuchi, one of the most severely damaged municipalities. OHRQoL and psychological distress were assessed using the General Oral Health Assessment Index (GOHAI) and the Kessler Psychological Distress Scale (K6), Japanese version, respectively. Among 11,411 residents, 1,987 returned the questionnaire (response rate, 17.4 %) and received an oral examination to determine number of present teeth, dental caries status, and tooth-mobility grade, and to assess periodontal health using the Community Periodontal Index. Relationships between GOHAI and related factors were examined by nonparametric bivariate and multinomial logistic regression analyses using GOHAI cutoff points at the 25^th^ and 50^th^ national standard percentiles.

**Results:**

GOHAI scores were significantly lower in the 50–69-age group compared with other age groups in this study and compared with the national standard score. In bivariate analyses, all factors assessed in this study (i.e., sex, age, evacuation from home, interruption of dental treatment, lost or fractured dentures, self-rated systemic health, serious psychological distress (SPD), economic status, number of teeth, having decayed teeth, CPI code, and tooth mobility) were significantly associated with OHRQoL. Subsequent multinomial logistic regression analyses revealed that participants of upper-middle age, who had received dental treatment before the disaster, who had lost or fractured dentures, and who had clinical oral health problems were likely to show low levels of OHRQoL. In addition, perceived systemic health and SPD were also related with OHRQoL.

**Conclusions:**

OHRQoL of disaster survivors was associated with oral problems stemming from the disaster in addition to factors related to OHRQoL in ordinary times such as clinical oral status and perceived systemic health. Furthermore, SPD was also associated with OHRQoL, which suggests the disaster’s great negative impact on both oral and mental health conditions.

## Background

On March 11, 2011, a huge earthquake (the Great East Japan Earthquake) with a measured magnitude of 9.0 was followed by a giant tsunami, which struck the northeastern part of Japan facing the Pacific Ocean. This disaster resulted in catastrophic damage to numerous towns and cities. Many administrative and medical functions in the affected communities were lost and most survivors were forced to live in shelters or temporary accommodations for long periods of time [1].

Oral health is important for eating, communication, and the comfort of survivors. However, water and food supplies, privacy for grooming, oral health care goods as well as other sanitary conditions were insufficient after the disaster [2]. Such substandard living conditions following the disaster likely reduced the oral health status of almost everyone. Therefore, it is essential to identify victim populations with poor oral health-related quality of life (OHRQoL) and provide them with adequate supports.

Previous research in ordinary times presented the risk factors for poor OHRQoL as objectively assessed oral conditions including “low number of teeth” [3–8] and “high number of decayed teeth” [5–7]. Other than oral-related issues, significant factors are reported to include age [6], female sex [7], certain markers of social status such as “low income” [6] and “low education levels” [3, 8], and low self-rating of general health [3, 5, 6, 8]. In addition to these factors, many survivors had unusual experiences affecting oral health such as lost or fractured dentures and interrupted dental treatment due to loss of their home dentists. Furthermore, psychological distress might be related to OHRQoL of disaster survivors because OHRQoL includes psychosocial aspects and psychological problems, both of which were prevalent in survivors of the Great East Japan Earthquake and tsunami [9].

However, at present, there are very few studies on post-disaster surveys of oral health, especially OHRQoL, using measurable scales [2, 10-12]. Therefore, 9 months after the Great East Japan Earthquake and tsunami, we conducted a questionnaire survey that included an OHRQoL measure, a psychological distress measure, and questions on disaster-related experiences as well as clinical surveys of survivors. The primary aim of the present study was to assess OHRQoL among survivors of the disaster. The secondary aim was to examine factors related to OHRQoL in disaster-affected areas.

## Methods

### Characteristics of the survey area

This survey was carried out in the town of Otsuchi, located on the Pacific Coast of Iwate Prefecture, which had suffered some of the most severe damage from the Great East Japan Earthquake and tsunami. Before the disaster, the main industries in Otsuchi were fisheries and fishery-related businesses such as processing and services. On March 11, 2011, the tsunami inundated primary administration, commerce, sightseeing, and inhabitable areas that were located primarily along the coastline. Because dental facilities were exclusively located in this area, all six dental offices in the town were destroyed. At the time of our survey, a temporary dental clinic had been established to provide dental services. The 2010 pre-disaster population of Otsuchi was 15,300. According to administrative records, the total number of dead and missing in the disaster was 1,311 (8.6 % of population).

### Study design and population

This was a cross-sectional study using questionnaire and clinical surveys performed in December 2011, 9 months after the disaster. At the time of the survey, we were uncertain of the post-disaster population due to various factors, including unconfirmed numbers of dead, missing, or relocated residents; therefore, we sent out notices of our health survey and questionnaire to all 11,411 residents aged 18 years or older based on the provisional figures available. Our survey was performed as part of a comprehensive health survey of survivors of the Great East Japan Earthquake and tsunami [9]. Furthermore, the comprehensive survey was done simultaneously with a health check-up examination that the town government performed as a public health service. Among 2,172 residents who received the town health check-up, 2,085 agreed to participate in the comprehensive survey. Thereafter, 74 declined to participate in the oral health examination. Among 2,001 participants in the oral health examination, we excluded 13 whose General Oral Health Index (GOHAI) score was missing. Finally, 1,987 participants (764 men and 1,223 women; mean age ± *SD*, 62.9 ± 14.1 and 60.4 ± 14.5 years, respectively) were the subjects in this study. Because Otsuchi consists of several smaller and geographically separate communities, to facilitate easy access, 11 health check-up venues were set up during the 15-day clinical survey period (December 8–22, 2011). At each venue’s health check-up reception area, we obtained participants’ informed consent to participate in our study.

The study protocol was approved by the Medical Ethics Committee of Iwate Medical University (H23-69) and conducted in accordance with the guidelines of the Declaration of Helsinki.

### Data collection

#### Self-reported data

We sent out a questionnaire and notice of our health survey to residents, and requested that they complete the questionnaire and bring it to their health check-up examination, at which time a trained interviewer was available to obtain more complete answers to any insufficient questionnaire responses.

We assessed OHRQoL using the Japanese version of the GOHAI [3, 4], which was originally developed for the elderly and called the Geriatric Oral Health Assessment Index. More recently, it is also known as the General Oral Health Assessment Index to reflect its applicability to younger age groups [5, 6, 13]. The GOHAI consists of 12 items measuring the degree of limitation in three domains: “physical function,” “pain and discomfort,” and “psychosocial function.” [14] The summary score of all 12 items ranges from 12 to 60 and represents an individual’s OHRQoL, with high summary scores indicating good OHRQoL.

We assessed psychological distress using the Kessler Psychological Distress Scale (K6), Japanese version [15, 16]. This questionnaire consists of six items measuring mental health on a 5-point Likert scale and asks respondents how often during the preceding 30 days they had felt (1) so sad that nothing could cheer them up; (2) nervous; (3) restless or fidgety; (4) hopeless; (5) worthless; and (6) that everything was an effort. Each item is scaled from 0 (none of the time) to 4 (all of the time). The total score of psychological distress is assessed by totaling the six item scores and ranges from 0 to 24. Contrary to the GOHAI, a high K6 score would indicate a low level of mental health (psychological distress). Based on previous studies, respondents with serious psychological distress (SPD) were identified by a score of 13 or higher [9, 15–21].

We created a questionnaire to obtain data on residents’ disaster-related experiences as well as their current systemic health and economic condition. In the questionnaire, respondents provided their current and pre-disaster residential addresses. If these differed, we categorized these respondents as having been evacuated from their home. Next, respondents were asked about interruption in dental treatment that they were receiving before the disaster. Answer choices were (1) “no dental treatment was being received before the disaster,” (2) “treatment was not interrupted,” (3) “treatment was interrupted and has resumed at the original dental clinic,” (4) “treatment was interrupted and has resumed at another dental clinic,” and (5) “dental care remains interrupted.” However, because we found no difference in GOHAI scores between choices (4), (5), and (6), we recategorized the choices for later analysis as follows: (1) “no dental treatment was being received before the disaster,” (2) “treatment was not interrupted,” and (3) “treatment was interrupted.” Next, we assessed the disaster’s impact on denture use according to the following criteria: (1) “did not wear dentures,” (2) “no disaster-related damage to dentures,” and (3) “disaster-related lost or fractured dentures.” Furthermore, we asked respondents for their self-rated systemic-health condition using four choices (very poor, poor, fair, or good) and current economic status also using four choices (very severe, severe, slightly severe, or normal). Based on their answers, we divided participants into three groups for self-rated systemic health (poor, fair or good) and two groups for economic status (severe, normal) in our analyses.

#### Clinical data

Oral examinations were performed by 4–6 skilled dentists per an examination day from Iwate Medical University School of Dentistry. All of them participated in preliminary meetings for this survey and received training for consistency in the assessments. Dental caries status was assessed according to the World Health Organization (WHO) method. In brief, examiners inspected each participant’s tooth under artificial lighting and recorded each tooth as sound, decayed, filled, or missing. Some modifications were made to the WHO method as follows: (1) a tooth with treated or untreated root caries was recorded as a filled or decayed tooth, and (2) a remaining root without a crown was counted as a present tooth. Periodontal conditions were assessed using the Community Periodontal Index (CPI) whose procedures and diagnostic criteria were also recommended by the WHO [22]. Briefly, using a special probe, examiners assessed three indicators of periodontal status: gingival bleeding, calculus, and periodontal pockets. Participants were divided by five codes of index-tooth severity (i.e., code 0 = healthy; code 1 = bleeding observed after probing the gingival sulcus; code 2 = calculus detected during probing; code 3 = periodontal pocket (4–5 mm); code 4 = periodontal pocket (6 mm or deeper). In addition, tooth-mobility grade based on CPI index teeth was recorded using Miller’s classification (i.e., 0 = normal mobility; grade 1 = slightly [<0.2 mm horizontal movement]; grade 2 = moderately [1–2 mm horizontal movement]; grade 3 = severe mobility [> 2 mm horizontal or any vertical movement]) [23].

### Statistical analysis

Because GOHAI scores were non-normally distributed, we used nonparametric tests for all analyses. For comparisons between our participants and comparisons with Japanese national standard scores by 10-year age groups, we used one-sample median tests. Simultaneously, we examined differences in GOHAI scores by age group using the Kruskal-Wallis test followed by multiple comparisons using the Mann–Whitney *U* test with Bonferroni correction. We also divided participants into three age groups (50, 50–69, and ≥ 70 years) for further factor analyses. First, we compared GOHAI according to participants’ characteristics obtained by questionnaire and clinical examination, using the Mann–Whitney *U* test to compare two groups, and the Kruskal-Wallis test to compare three or more groups. When the Kruskal-Wallis test revealed significant differences, we conducted multiple comparisons using the Mann–Whitney *U* test with Bonferroni correction. Next, we divided GOHAI scores into three ranges using 25^th^ (48.7 points) and 50^th^ (55 points) percentiles of the national standard score, which was determined using data on 3,283 subjects from 26 dental facilities located in widely different areas of Japan in 2006 [3, 4, 24]. Therefore, we identified participants with GOHAI scores ranging from 49 to 54 as individuals with poor OHRQoL and those with GOHAI scores of 48 or less with very poor OHRQoL. Against the reference group (GOHAI ≥ 55), we assessed measurement risks by multinomial logistic regression models, and those independent variables were adjusted for each variables. In the multiple models based on single correlation analyses, “interruption of dental treatment” and “CPI code” were recategorized as the binary variables “receiving dental treatments before disaster (0 = no; 1 = yes)” and “CPI code 4 (0 = no, yes = 1).” Two-sided *p*-values less than 0.05 were considered statistically significant. All statistical analyses were conducted using the software program SPSS version 19.0 for Windows (IBM).

## Results

### GOHAI score distribution

**Fig. 1:**
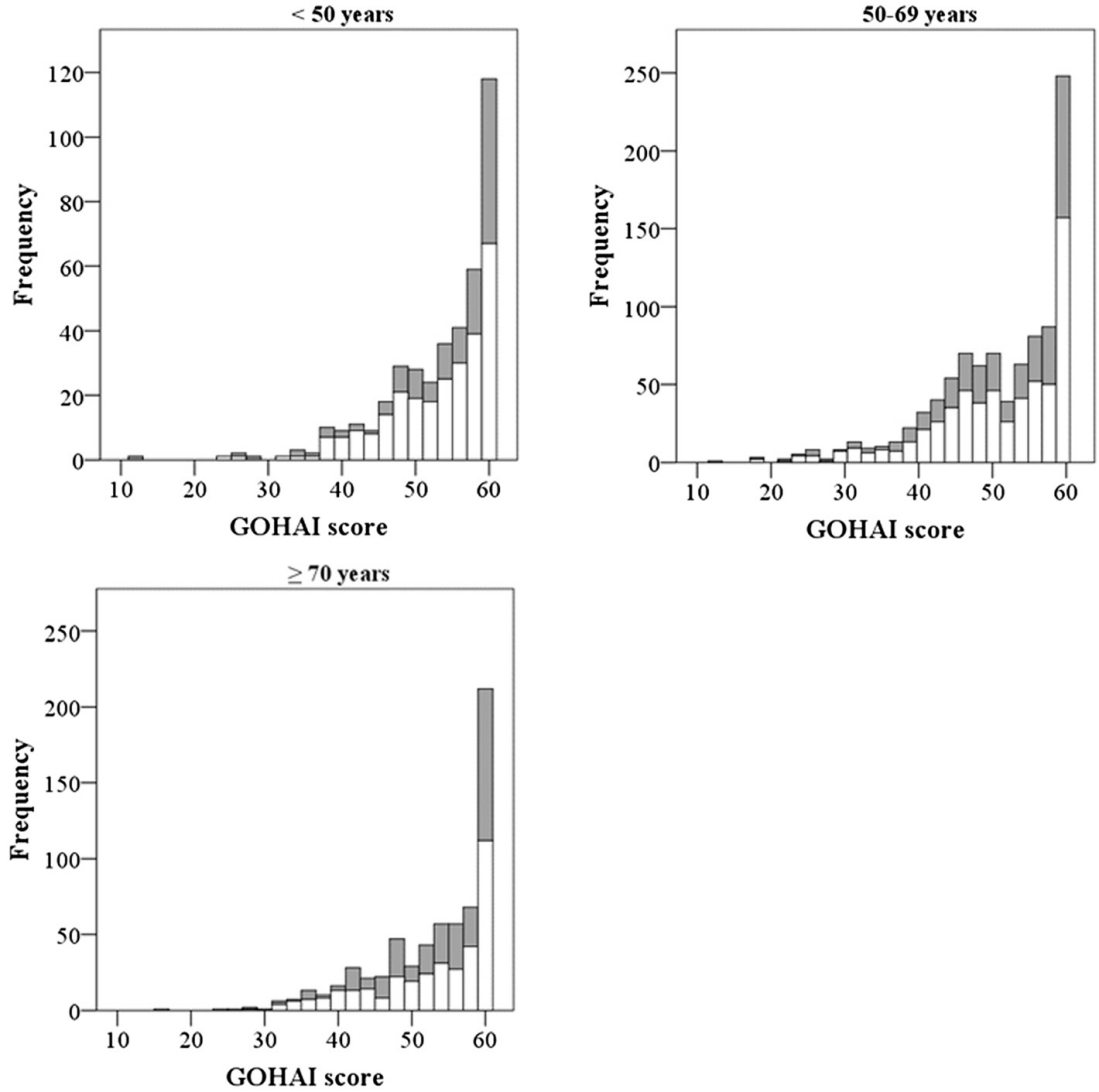
GOHAI score distributions separated by three age groups expressed as histograms. Bar indicates number of participants (gray square men, white square women). Mean (*SD*) GOHAI score in each age class was as follows: 53.1 (7.5) for < 50 years, 51.0 (8.8) for 50–69 years, and 52.8 (7.9) for > 70 years

Table 1 shows numbers of subjects by age and sex. In this study, subjects represented 17.3 % of the total population. The sex ratio (male/female) of participants was 0.62, which was significantly lower than the sex ratio of 0.82 in the total population (*p* < 0.01 by Chi-squared test). Table 2 summarizes GOHAI-score distributions by 10-year age groups compared with the national standard in Japan. The Kruskal-Wallis test revealed a significant difference in average rank of GOHAI score by subject’s age group. The lowest score was in the 50–59-age group and the next lowest score was in the 60–69-age group. Post hoc multiple comparisons using the Mann–Whitney *U* test with Bonferroni correction showed that these two groups exhibited significantly lower scores than the other age groups. Those groups as well as the 40–49-age group showed a significantly lower median score than the national standard. In contrast, the median of the 70–79-age group was significantly higher than the national standard. We conducted the following factor analysis in the respective age groups divided as follows: < 50 years, 50–69 years, and ≥ 70 years. Figure 1 presents histograms of GOHAI-score distributions by the three age groups, and every non-normal GOHAI distribution was confirmed by one-sample Kolmogorov-Smirnov tests (all *p* < 0.001).

**Table 1:** Numbers of subjects by age and sex

		Age group (in years)							Total
		18–29	30–39	40–49	50–59	60–69	70–79	≥ 80	
Men	No. of subjects	21	47	66	97	245	235	53	764
	Population	628	689	792	1,019	1,118	798	366	5,410
	Percent	3.3	6.8	8.3	9.5	21.9	29.4	14.5	14.1
	Mean age ± *SD*	24.5 ± 4.2	35.2 ± 2.7	44.2 ± 2.7	55.1 ± 2.8	64.9 ± 2.7	73.9 ± 2.8	82.2 ± 2.0	62.9 ± 14.1
Women	No. of subjects	37	100	132	195	405	277	77	1,223
	Population	635	603	812	953	1,176	1,131	777	6,087
	Percent	5.8	16.6	16.3	20.5	34.4	24.5	9.9	20.1
	Mean age ± *SD*	24.9 ± 3.2	34.4 ± 2.8	44.6 ± 3.8	55.1 ± 2.8	64.3 ± 2.9	73.8 ± 2.6	82.6 ± 2.7	60.4 ± 14.5
Total	No. of subjects	58	147	198	292	650	512	130	1,987
	Population	1,263	1,292	1,604	1,972	2,294	1,929	1,143	11,497^a^
	Percent	4.6	11.4	12.3	14.8	28.3	26.5	11.4	17.3
	Mean age ± *SD*	24.6 ± 3.7	34.7 ± 2.8	44.4 ± 3.4	55.1 ± 2.8	64.5 ± 2.9	73.8 ± 2.7	82.4 ± 2.4	61.3 ± 14.4

^a^Definite number in October, 2011 (source: government of Otsuchi), which is slightly different from the provisional number (11,411) used for distribution of notices of our survey

**Table 2:** Distribution of GOHAI score by age

	Age group (in years)							Total (*N* = 1987)
	18–29	30–39	40–49	50–59	60–69	70 –79	≥ 80	
Range	25–60	33–60	12–60	12–60	18–60	16–60	28–60	12–60
	34–60	34–60	30–60	25–60	31–60	25–60	N. A.	25–60
Mean ± *SD*	54.7 ± 7.1	53. 8 ± 6.9	52.1 ± 7.9	50.2 ± 9.2^a^	51.4 ± 8.6^b^	52.8 ± 7.8	52.7 ± 8.4	52.0 ± 8.3
	53.3 ± 6.5	54.3 ± 6.5	53.7 ± 6.8	52.2 ± 7.8	52.6 ± 7.2	50.8 ± 8.8	N. A.	53.1 ± 7.0
Median	58.0	56.0	54.0^c^	52.0^c^	53.0^c^	55.0^d^	56.0	54.0^c^
	55.0	55.5	55.7	54.2	54.2	52.8	N.A.	55.0
25^th^ percentile	50.8	50.0	47.8	46.0	46.0	48.0	47.8	47.0
	49.5	51.0	50.4	46.5	47.0	45.3	N. A.	48.7
75^th^ percentile	60.0	60.0	58.0	59.0	59.0	60.0	60.0	60.0
	59.1	59.9	59.5	59.0	59.0	58.8	N. A.	59.3

*Note*. Upper values show survey results and lower values represent the 2006 national standard in Japan

*NA* Not applicable because the study for the 2006 national standard did not target this age class

^a^Significantly lower than 20s (*p* = 0.002), 30s, 70s, and 80s (all *p* < 0.001) by multiple comparisons following Kruskal-Wallis test between age groups

^b^Significantly lower than 20s (*p* = 0.029) by multiple comparisons following Kruskal-Wallis test between age groups

^c^Significantly lower median than national standard (*p* < 0.001)

^d^Significantly lower median than national standard (*p* = 0.016)

### Bivariate relationships of GOHAI scores with measurements

#### Relationship between GOHAI score and sex, age and self-reported data

There were significant differences in GOHAI score by all assessed items (Table 3). Subsequent multiple comparisons were carried out for “age,” “interruption of dental treatment,” “lost or fractured dentures,” and “self-rated systemic health.” As a result, the 50–69-age group was found to have significantly lower GOHAI score than the other two groups. Among the three response groups for “interruption of dental treatment,” respondents of “no dental treatment was being received before the disaster” had significantly higher GOHAI scores than the other two groups, whereas we found no difference between respondents of “treatment was not interrupted” and “treatment was interrupted and has resumed at the original dental clinic.” For “lost or fractured dentures,” those who responded “yes” had significantly lower GOHAI scores than the other groups. As for “self-rated systemic health,” those whose self-rating was “good” had significantly higher GOHAI scores than the other two groups. In addition, those whose self-rating was “fair” had higher GOHAI scores than those whose self-rating was “poor.”

**Table 3:** Difference in GOHAI score by sex, age, and disaster-related experiences

	No. subject	Mean ± *SD*	*p*-value
Sex			
Men	764	52.5 ± 8.2	0.04
Women	1,223	51.7 ± 8.3	
Age (in years)			
< 50 years	403	53.1 ± 7.5	< 0.01
50–69 years	942	51.0 ± 8.8	
≥ 70 years	642	52.8 ± 7.9	
Evacuation from home			
No	687	52.6 ± 7.9	0.02
Yes	1,300	51.7 ± 8.8	
Interruption of dental treatment			
Not receiving	1,619	52.7 ± 7.8	< 0.01
Not interrupted	37	48.7 ± 8.8	
Interrupted	317	49.0 ± 9.6	
Lost or fractured dentures			
Do not wear dentures	815	52.5 ± 8.2	< 0.01
No damage	985	52.2 ± 8.2	
Yes	172	48.8 ± 8.9	
Self-rated systemic health			
Good	198	56.2 ± 5.8	< 0.01
Fair	1,463	52.3 ± 7.9	
Poor/very poor	322	48.0 ± 9.9	
SPD			
No (K6 score < 13)	1,311	53.5 ± 7.2	< 0.01
Yes (K6 score ≥ 13)	640	49.1 ± 9.6	
Economic status			
Fair	973	52.7 ± 5.8	< 0.01
Severe	1,014	51.4 ± 7.9	

#### Relationship between GOHAI scores and current oral conditions

We also found significant differences in all measurements of assessed oral health conditions (Table 4). In subsequent multiple comparisons, subjects with 1–19 teeth had significantly lower GOHAI scores than other subjects, whereas we found no difference between edentulous subjects and subjects with 20 or more teeth. GOHAI scores of subjects with CPI code 4 were significantly lower than those of subjects with CPI code 0–2 and subjects with no index teeth. Furthermore, subjects with moving teeth at grade 2 or 3 had significantly lower GOHAI scores than the other subjects.

**Table 4:** Difference in GOHAI score for objectively assessed oral conditions

	No. of subjects	Mean ± *SD*	*p*-value
Number of present teeth			
Edentulous	352	52.9 ± 8.0	< 0.01
1–19	658	50.2 ± 9.1	
20 or more	977	52.9 ± 7.6	
Having one or more decayed teeth			
No	1,259	52.7 ± 7.8	< 0.01
Yes	728	50.9 ± 9.0	
CPI code			
No index teeth	447	52.4 ± 8.2	0.01
0–2	818	52.4 ± 7.9	
3	517	51.7 ± 8.5	
4	205	50.3 ± 8.3	
Tooth-mobility grade			
No index teeth	447	52.4 ± 8.2	< 0.01
0–1	1,374	52.2 ± 8.3	
2 or 3	167	49.3 ± 8.6	

### Multinomial logistic regression of OHRQoL

Multinomial logistic regression analyses were performed for 1,964 subjects without any missing values. In the discrimination models for OHRQoL levels, “sex,” “evacuation from home,” “economic status,” and “CPI code 4,” we found no significant adjusted odds ratio (AOR) for both poor and very poor OHRQoL status. As Table 5 shows, variables significantly related to both very poor and poor OHRQoL status, respectively, were “receiving dental treatments before disaster” (AOR, 2.13; 95 % confidence interval (CI), 1.59–2.85 and AOR, 1.44; 95 % CI, 1.04–1.99); “lost or fractured dentures” (AOR, 2.32; 95 % CI, 1.54–3.50 and AOR, 1.99; 95 % CI, 1.27–3.14); “self-rated systemic-health” (AOR, 6.15; 95 % CI, 3.58–10.6 and AOR, 2.46; 95 % CI: 1.45–4.17 for “poor/very poor,” and AOR, 3.03; 95 % CI, 1.88–4.87 and AOR,1.97; 95 % CI: 1.29–3.00 for “fair” vs. “good”); and “having one or more decayed teeth” (AOR, 1.54; 95 % CI, 1.20–1.97 and AOR, 1.36; 95 % CI: 1.04–1.77).

**Table 5:** Multinomial logistic regression of OHRQoL

	OHRQoL						
	Very poor (GOHAI ≤ 48)			Poor (49 ≤ GOHAI < 55)			Reference No. (GOHAI ≥ 55)
	*n*	COR (95 % CI)	AOR (95 % CI)	*n*	COR (95 % CI)	AOR (95 % CI)	
		*p*-value	*p*-value		*p*-value	*p*-value	
Sex							
Women	377	1.08 (0.99–1.49)	1.11 (0.40–1.40)	262	1.08 (0.99–1.18)	1.21 (0.94–1.56)	392
		0.09	0.40		0.12	0.13	
Men^a^	215			148			570
Age (in years)							
≥ 70	174	1.06 (0.95–1.19)	1.27 (0.86–1.87)	129	0.99 (0.88–1.13)	1.27 (0.68–1.50)	334
		0.32	0.23		0.94	0.31	
50–69	322	1.18 (1.09–1.27)	1.68 (1.28–2.20)	196	1.07 (0.97–1.17)	1.24 (0.93–1.66)	411
		< 0.01	< 0.01		0.22	0.06	
< 50	96			85			217
Evacuation from home							
Yes	200	1.05 (0.97–1.13)	1.02 (0.81–1.30)	128	0.85 (0.72–1.00)	0.83 (0.64–1.08)	353
		0.25	0.86		0.06	0.16	
No	392			282			609
Receiving dental treatments before disaster							
Yes	442	1.98 (1.60–2.46)	2.13 (1.59–2.85)	322	1.49 (1.15–1.93)	1.44 (1.04–1.99)	839
		< 0.01	< 0.01		< 0.01	0.03	
No	150			78			123
Lost or fractured dentures							
Yes	79	2.57 (1.83–3.60)	2.32 (1.54–3.50)	41	1.92 (1.29–2.86)	1.99 (1.27–3.14)	50
		< 0.01	< 0.01		< 0.01	< 0.01	
No	513			369			912
Self-rated systemic health							
Poor/very poor	152	2.05 (1.75–2.39)	6.15 (3.58–10.6)	62	1.59 (1.30–1.95)	2.46 (1.45–4.17)	104
		< 0.01	< 0.01		< 0.01	< 0.01	
Fair	415	1.13 (1.09–1.18)	3.03 (1.88–4.87)	318	1.09 (1.05–3.00)	1.97 (1.29–3.00)	715
		< 0.01	< 0.01		< 0.01	< 0.01	
Good	23			30			141
SPD (K6 ≥ 13)							
Yes	274	1.97 (1.71–2.27)	2.32 (1.82–2.95)	126	1.30 (1.09–1.57)	1.28 (0.97–1.68)	228
		< 0.01	< 0.01		< 0.01	0.08	
No	304			276			720
Economic status							
Severe	325	1.13 (1.03–1.25)	1.17 (0.93–1.46)	198	1.07 (0.95–1.20)	0.90 (0.88–1.42)	496
		0.01	0.19		0.27	0.37	
Normal	267			212			466
Number of present teeth							
Edentulous	94	1.04 (0.84–1.28)	1.75 (1.19–2.55)	66	0.88 (0.69–1.12)	1.03 (0.69–1.54)	188
		0.74	< 0.01		0.28	0.88	
1**–**19	255	1.51 (1.32–1.71)	1.91 (1.44–2.53)	130	1.11 (0.94–1.32)	1.08 (0.80–1.46)	263
		< 0.01	< 0.01		0.22	0.62	
20 or more	243			214			511
Having one or more decayed teeth							
Yes	343	1.30 (1.14–1.80)	1.54 (1.20–1.97)	251	1.20 (1.03–1.39)	1.36 (1.04–1.77)	650
		< 0.01	< 0.01		0.03	0.02	
No	249			159			312
CPI code 4							
Yes	78	1.58 (1.18–2.13)	1.43 (0.97–2.11)	44	1.29 (0.91–1.83)	1.35 (0.89–2.07)	80
		< 0.01	0.08		0.18	0.16	
No	514			366			882
Having one or more moving teeth at grade 2 or 3							
Yes	79	2.21 (1.60–3.06)	1.73 (1.14–2.64)	27	1.09 (0.70–1.70)	0.89 (0.53–1.49)	58
		< 0.01	0.01		0.71	0.66	
No	513			383			904

*Note. COR* crude odds ratio, *AOR* adjusted odds ratio, *CI* confidence interval

^a^The last row shows the reference category for each explanatory variable

Variables exclusively associated with very poor OHRQoL status were SPD (K6 ≥ 13) (AOR, 2.32; 95 % CI, 1.82–2.95), “number of present teeth” (AOR, 1.91; 95 % CI, 1.44–2.53 for “1–19” and AOR, 1.75; 95 % CI, 1.19–2.55 for “edentulous” vs. “20 or more”), and “having one or more moving teeth at grade 2 or 3” (AOR, 7.73; 95 % CI, 1.14–2.64).

## Discussion

This is the first study to adapt the GOHAI to survivors of a huge disaster. We found that GOHAI score was significantly lower in all participants compared with the median national standard score. By 10-year age group, GOHAI scores were significantly lower in the 40–49-, 50–59- and 60–69-age groups in this study compared with the national standard scores. Furthermore, GOHAI scores were significantly lower in the 50–59- and 60–69-age groups than in the other 10-year age groups in this study (Table 2). After dividing participants into three age groups, GOHAI score was found to be significantly lower in the 50–69 age group than in the other age groups by bivariate and multivariate analyses (Tables 3 and 5). Some earlier studies using the GOHAI Japanese version, as in the present study, have reported no remarkable age-related differences in scores [3–5]. Hence, the lower GOHAI score of disaster survivors particularly among the upper-middle age group, might be characteristic of victims from a disaster area. As one of the reasons, we speculate that these participants, as the head of their family, were forced to spend most of their time doing extraordinary things to survive after the disaster. Consequently, they could not take care of their own oral health.

For multinomial logistic regression of the GOHAI, we used 25^th^ and 50^th^ percentiles of the national standard as cutoff values. Previous studies have used several cutoff points for discrimination of low GOHAI groups. To assess dental care needs in ordinary times, relatively high cutoff points were previously used (54 to 58 points) [5, 8, 25], whereas researchers who attempted to identify subjects with high dental needs or to survey diabetes patients used low 25^th^ percentile scores (40 or 44 points) as cutoff values [7, 26]. To identify residents with both very poor and poor OHRQoL status, we used two cutoff values, those at the 25^th^ and 50^th^ percentiles of the national standard, respectively. As a result, no measurement was exclusively related to a poor level of OHRQoL. On the contrary, “age,” SPD, “number of present teeth,” and “having one or more moving teeth at grade 2 or 3” exclusively showed a significant AOR versus the very poor OHRQoL level (Table 5). These findings suggest that the 25^th^ percentile we used is a suitable cutoff value to survey OHRQoL of residents in a disaster victim area.

Results of the questionnaire survey indicated that disaster-related experiences concerning oral health problems such as “receiving dental treatments before disaster” and “lost or fractured dentures” degraded OHRQoL levels. Both relationships with OHRQoL were naturally expected because the subjects who had received dental treatment before the disaster or who were deprived of their dentures had high dental needs, especially under conditions of scarce dental resources. Although our survey was carried out 9 months after the disaster, 70 % of participants with lost or fractured dentures had already had them restored or repaired primarily by temporary dental care services set up for tsunami victims. However, their GOHAI scores did not differ from those participants whose dentures were not restored or repaired (data not shown). Removable dentures usually require periodic adjustments. Kivovics et al. reported that 87 % of complete new dentures required at least one adjustment [27]. In addition, Veyryne et al. showed that GOHAI scores of subjects who received new prostheses showed improvement after 12 weeks even in ordinary times [28]. Thus, scarce dental resources after a disaster can limit adequate adjustments after denture re-restoration or repair and continue to suppress OHRQoL levels in disaster areas. The need for continuous dental care support was suggested by our results showing no difference in GOHAI scores between those whose dental treatments were interrupted and those whose treatments were not interrupted (Table 3), which also indicates insufficient post-disaster dental care. Thus, continuous dental care appears to be needed for people in disaster areas.

The objectively assessed oral conditions found to be related to a very low GOHAI score were “number of present teeth” and “having one or more decayed teeth,” which agreed with several previous studies [1–6, 29]. Although the relationship between tooth mobility and OHRQoL has been rarely reported, Ng and Leung reported the relationship between self-reports of “having drifting teeth” and OHRQoL measured by the Oral Health Impact Profile (OHIP-14) [30, 31]. The OHIP-14 mean score, where a high score indicates low OHRQoL, was remarkably higher in subjects who reported “having drifting teeth” than those who did not, although the difference was not significant. People are naturally expected to easily perceive tooth mobility as an oral health problem. In contrast, “CPI code 4” did not show a significant AOR with very poor OHRQoL levels, although a significant crude odds ratio was observed (Table 5). A tooth recorded as CPI code 4 would be likely to show a high grade of mobility, which might have confounded the two measures. In addition, as Zaitsu et al. previously indicated [5], CPI criteria consisting of bleeding on probing, dental calculus, and periodontal pockets might be difficult for the general public to recognize. Thus, tooth mobility might be a more sensitive measure to assess OHRQoL than the examination using CPI.

Multinomial analyses showed that “self-rated systemic health” and SPD was significantly related with very poor OHRQoL among measures other than oral-related issues. It is interesting that OHRQoL levels were associated with health domains more strongly than socioeconomic aspects such as “evacuation from home” and “economic status.” Relationship of OHRQoL with “self-rated systemic health” as well as “socioeconomic status” has been reported [3, 5, 6, 8] in ordinary situations. Whereas the association between oral health status and psychological problems was unclear, a few studies have suggested the relationship [21, 32, 33]. However, our results clearly indicate that a relationship exists. The experience of disaster followed by substandard living conditions might lead to various levels of impact according to survivors’ mental and physical properties. Compared with physical and mental problems, social problems would not be a concern because many victims lived together under similar circumstances no matter how terrible it was. Hence, we supposed that the great impact of a disaster on both mental and physical health, including oral health, accounted for the clearly observed relationship between mental and oral health status.

This study had several limitations. First, our participants were not probability samples because they voluntarily participated in systemic-health check-up services conducted by the town government. Furthermore, the proportion of young and male participants was relatively low because such survivors who had some form of employment at the time were probably unable to join our study. Nevertheless, we believe our participants represented all residents to a noteworthy degree because their number exceeded the required numbers with 95 % confidence and a 5 % margin of error for both men and women (764 vs. 359 for men, and 1,223 vs. 361 for women). Second, oral examinations were performed by multiple examiners under trying conditions at facilities in the disaster area. This might result in greater interobserver variability than under ordinary conditions. Third, being a cross-sectional study, it necessarily lacked data from the affected region before the disaster, although we expect to provide supplemental data in a follow-up study. Finally, whether our findings are specific to a disaster area or not remains unclear given the lack of comparison data for nondisaster situations. However, we consider our results valuable for identifying relative post-disaster risks of oral health because we mainly analyzed internal correlates in a group of survivors.

## Conclusion

OHRQoL of survivors living in a victim area was lower than the national standard, especially in the 50–69-age group. Their OHRQoL was reduced by objectively worse oral condition and negatively perceived systemic health condition, which were also observed in ordinary times. In addition to these factors, disaster-related oral problems, such as being deprived of dentures and dental treatment that had existed before the disaster, remarkably degraded their OHRQoL levels. Furthermore, SPD was also associated with OHRQoL, which suggests that disaster experiences have a great negative impact on both oral and mental health conditions. Our study strongly indicates that continuous dental care support as well as mental health care is needed for survivors of a huge disaster.

## Abbreviations

OHRQoL: Oral Health-Related Quality of Life
GOHAI: General Oral Health Assessment Index
CPI: Community Periodontal Index
SD: Standard deviation
CI: Confidence interval
COR: Crude odds ratio
AOR: Adjusted odds ratio
OHIP: Oral Health Impact Profile
SPD: Serious psychological distress.

## References

[1] Nohara M (2012). Impact of the Great East Japan Earthquake and tsunami on health, medical care and public health systems in Iwate Prefecture, Japan, 2011. Western Pac Surveill Response J..

[2] Hosokawa R, Taura K, Ito E, Koseki T (2012). Roles of dentists and dental hygienists in two major earthquakes. Int Dent J..

[3] Naito M, Suzukamo Y, Nakayama T, Hamajima N, Fukuhara S (2006). Linguistic adaptation and validation of the General Oral Health Assessment Index (GOHAI) in an elderly Japanese population. J Public Health Dent..

[4] Naito M, Suzukamo Y, Nakayama T, Fukuhara S (2004). Preliminary study on the development of an oral health-related QOL scale: production of a Japanese version of the General Oral Health Assessment Index (GOHAI). J Dent Health..

[5] Zaitsu T, Ueno M, Shinada K, Ohara S, Wright FA, Kawaguchi Y (2011). Association of clinical oral health status with self-rated oral health and GOHAI in Japanese adults. Community Dent Health.

[6] Tubert-Jeannin S, Riordan PJ, Morel-Papernot A, Porcheray S, Saby-Collet S (2003). Validation of an oral health quality of life index (GOHAI) in France. Community Dent Oral Epidemiol..

[7] El Osta N, Tubert-Jeannin S, Hennequin M, Bou Abboud Naaman N, El Osta L, Geahchan N (2012). Comparison of the OHIP-14 and GOHAI as measures of oral health among elderly in Lebanon. Health Qual Life Outcomes.

[8] Fuentes-García A, Lera L, Sánchez H, Albala C (2013). Oral health-related quality of life of older people from three South American cities. Gerodontology..

[9] Yokoyama Y, Otsuka K, Kawakami N, Kobayashi S, Ogawa A, Tanno K (2014). Mental health and related factors after the Great East Japan Earthquake and Tsunami. PLoS One.

[10] Liu D, Hu D, Li X, Ma H (2010). Periodontitis in 65-74-year-old victims in Wenchuan, China postearthquake: implications for service provision. Int Dent J..

[11] Karim A, Wager B, Khan AA (2009). Caries prevalence in Chikar, Kashmir, post-earthquake: implications for service provision. Int Dent J..

[12] Sato Y, Aida J, Takeuchi K, Ito K, Koyama S, Kakizaki M (2015). Impact of loss of removable dentures on oral health after the Great East Japan Earthquake: a retrospective cohort study. J Prosthodont..

[13] Atchison KA, Dolan TA (1990). Development of the Geriatric Oral Health Assessment Index. J Dent Educ..

[14] Atchison KA, Der-Martirosian C, Gift HC (1998). Components of self-reported oral health and general health in racial and ethnic groups. J Public Health Dent..

[15] Kessler RC, Andrews G, Colpe LJ, Hiripi E, Mroczek DK, Normand SL (2002). Short screening scales to monitor population prevalences and trends in non-specific psychological distress. Psychol Med..

[16] Furukawa TA, Kawakami N, Saitoh M, Ono Y, Nakane Y, Nakamura Y (2008). The performance of the Japanese version of the K6 and K10 in the World Mental Health Survey Japan. Int J Methods Psychiatr Res..

[17] Sakurai K, Nishi A, Kondo K, Yanagida K, Kawakami N (2011). Screening performance of K6/K10 and other screening instruments for mood and anxiety disorders in Japan. Psychiatry Clin Neurosci..

[18] Kessler RC, Barker PR, Colpe IJ, Epstein JF, Gfroerer JC (2003). Screening for serious mental illness in the general population. Arch Gen Psychiatry..

[19] Drapeau A, Marchand A, Forest C (2014). Gender differences in the age-cohort distribution of psychological distress in Canadian adults: findings from a national longitudinal survey. BMC Psychology..

[20] Suzuki T, Miyaki K, Sasaki Y, Song Y, Tsutsumi A, Kawakami N (2014). Optimal cutoff values of WHO-HPQ presenteeism scores by ROC analysis for preventing mental sickness absence in Japanese prospective cohort. PLoS One..

[21] Xiang X, Lee W, Kang SW (2015). Serious psychological distress as a barrier to dental care in community-dwelling adults in the United States. J Public Health Dent..

[22] World Health Organization (1997). Oral health surveys: basic methods.

[23] Laster L, Laudenbach KW, Stoller NH (1975). An evaluation of clinical tooth mobility measurements. J Periodontol..

[24] Naito T (2006). **A**ssessment of Oral Health Related QOL—Significance and Summary of Baseline Survey. J Health care Dent..

[25] Zuluaga DJ, Montoya JA, Contreras CI, Herrera RR (2012). Association between oral health, cognitive impairment and oral health-related quality of life. Gerodontology..

[26] Nikbin A, Bayani M, Jenabian N, Khafri S, Motallebnejad M (2014). Oral health-related quality of life in diabetic patients: comparison of the Persian version of Geriatric Oral Health Assessment Index and Oral Health Impact Profile: A descriptive-analytic study. J Diabetes Metab Disord.

[27] Kivovics P, Jáhn M, Borbély J, Márton K (2007). Frequency and location of traumatic ulcerations following placement of complete dentures. Int J Prosthodont..

[28] Veyrune JL, Tubert-Jeannin S, Dutheil C, Riordan PJ (2005). Impact of new prostheses on the oral health related quality of life of edentulous patients. Gerodontology..

[29] Gerritsen AE, Allen PF, Witter DJ, Bronkhorst EM, Creugers NH (2010). Tooth loss and oral health-related quality of life: a systematic review and meta-analysis. Health Qual Life Outcomes..

[30] Ng SK, Leung WK (2006). Oral health-related quality of life and periodontal status. Community Dent Oral Epidemiol.

[31] Slade GD (1997). Derivation and validation of a short form of the oral health impact profile. Community Dent Oral Epidemiol..

[32] Amarasena N, Kapellas K, Brown A, Skilton MR, Maple-Brown LJ, Bartold MP (2015). Psychological distress and self-rated oral health among a convenience sample of Indigenous Australians. J Public Health Dent..

[33] Sánchez-García S, Heredia-Ponce E, Juárez-Cedillo T, Gallegos-Carrillo K, Espinel-Bermúdez C, de la Fuente-Hernández J (2010). Psychometric properties of the General Oral Health Assessment Index (GOHAI) and dental status of an elderly Mexican population. J Public Health Dent..

